# The New York Odontological Society

**Published:** 1885-01

**Authors:** 


### THE NEW YORK ODONTOLOGICAL SOCIETY.

### NOTES OF AN OBSERVER.

At the November meeting of the New York Odontological Society Dr. N. W. Kingsley read from copies of the *Medical Record* a number of articles, including editorials, on the subject of "dead teeth," and their influence on health when retained in the mouth. Every contributor who had written on the subject gave expression to the belief that diseased dental organs were a frequent cause of dire mischief to the animal economy, and some of the writers even intimated that roots and pulpless teeth should never be permitted to remain in the mouth, believing that although no perceptible injury might be manifested, or pain occasioned by their presence, they were nevertheless sources of constant local irritation, and produced constitutional disturbances which were likely to result in serious derangement to the body corporeal.

Many cases were noted from published reports of Dr. Samuel Sexton, Surgeon-in-Chief of the N. Y. Ear Dispensary, citing cases where diseases of the ear were supposed to have been occasioned by the presence of devitalized molars. In some of these cases a cure was effected by simply extracting one or more of these suspicious members.

The so-called "bridge-work," where dead teeth and roots are utilized as supports for artificial dentures, was severely criticised by one of the writers, who emphatically condemned this style of practice. Several of the contributors questioned the practicability of attempting the treatment of alveolar abscess, believing it an operation so extremely difficult to perform that few dentists existed who are competent to undertake the task, and even with good treatment such teeth were again likely to create trouble. Other writers did not consider such operations so very difficult, and believed that a large number of dentists treated diseased pulps, alveolar abscess and pulpless roots successfully, thereby saving them for long periods of usefulness, and with no discomfort to their possessors.

In commenting on these articles Dr. Kingsley could see much that he approved ; but some of the assertions he considered ridiculous. He could, however, see sense in a paragraph from one of the editorials, which suggested that physicians in general practice should become better posted in oral diseases.

*Dr. J. Morgan Howe*—the author of one of the papers published in the *Medical Record,* stated that he fully believed that dental lesions were important factors in the origin of many diseases, and were frequently the cause of much mischief. He spoke of exostosis, pericementitis, alveolar abscess, etc., as among these evils. He could not agree with a statement of Dr. Sexton, that "pulpless teeth can never remain in the mouth without causing irritation and endangering health." He can give numerous instances where they have been successfully treated, and has in his own mouth three pulpless teeth which were well treated, and for years have done their work faithfully, and are as comfortable as any of their co-laborers in the dental arches. He knew that a great many dentists were competent to do such work, and to do it well; yet care and skill were necessary in these cases. Dr. Howe further stated that the practice of indiscriminately capping pulps in their various conditions of health or disease was productive of more mischief and suffering than that induced by treating pulpless teeth. He also referred to the nourishment teeth receive through the pericementum, which keeps in a healthy condition many teeth deprived of their pulps.

*Prof. Frank Abbott*—author of another of the articles quoted from the *Medical Record,* dilated at length on the relation of diseased teeth to the general health. He thought Dr. Sexton deserved much credit for giving this subject so much attention, and believed his investigations would result in much good. He coincided with many of the views thus expressed, and he also had witnessed much trouble from a retention of worthless teeth rocking in diseased sockets, from alveolar abscess and pyorrhœa alveolaris. He reminded his hearers of the fact that immense numbers of diseased teeth are never presented to dentists for treatment, and that many are extracted in dispensaries, hospitals, and other places.

*Dr. H. C. Houghton*—A physician connected with the Ophthalmic Hospital instanced a visit to a lunatic asylum, through the apartments of which he was conducted by one of the inmates, whose mind, however, on some points was apparently clear. In calling the doctor's attention to a certain hobby of a fellow "lunatic," he quaintly remarked that there was simply this difference between the inmates and their visitors—"the former rode hobbies within the walls, while the latter rode them outside." The doctor seemed to realize the pertinency of the suggestion. He, however, thought the subject an important one, and was much interested in the articles read by Dr. Kingsley, and the discussion which followed. He could fully agree with the statement of Dr. Howe regarding the capping of pulps. He had also witnessed trouble from teeth over-filled, or fillings so prominent as to prevent the teeth from coming naturally together, thus permitting too much force to come on a single point.

DECEMBER MEETING.

This was held at the residence of Dr. Wm. H. Dwinelle, on the evening of the 16th.

The merits of cocaine were discussed by a number of gentlemen present, who related each his individual experience with this pain-obtunding agent, in operations within the oral cavity.

The opinion seemed to prevail that it had little, if any, effect on sensitive dentine, but in cases of pulpitis its employment had been marked with various degrees of success, but perhaps not more so than with other agents in general use. It was also claimed that it can be used with perfect safety, and has proved an effective anæsthetic when applied to soft tissue; but its action on hard tissue is somewhat imaginary. It was also stated that experiment had demonstrated that a four per cent solution of cocaine was sufficient for all practical purposes, and no better result would follow if the solution was much stronger.

*Dr. W. Geo. Beers*—of Montreal, read a very interesting paper on "The Treatment of Children's Teeth." It embodied an earnest plea for the little ones who, as a rule, suffer much from the fruits of neglect, and whose teeth at an early age show evidences of disease, and are kept in a condition ever favorable to attacks from caries. Considering the abuse to which human teeth are subjected, instead of wondering why they decay so soon, it is a wonder how they can last so long. The lack of nourishment during the embryotic period, the want of better nourishment in infancy and through childhood, a too free indulgence in the use of saccharine medleys, were cited as among the predisposing causes of premature decay. He would give children a proper quantity of food, rich in lime phosphates. Can see no reason why preparations of phosphates of lime should not be of benefit to children with impoverished teeth. If well digested and assimilated, a sufficient amount would go to supply the requirements of the animal frame-work, and if expectant mothers would keep themselves properly nourished, and practically observe hygienic laws, they would bring into the world children showing a better condition of health, and with prospects of good teeth, which if kept in a condition of cleanliness would serve them for many years.

*Dr. F. W. Seabury*—of Providence, R. I., exhibited a new vulcanizer, a beautifully constructed apparatus of his own invention, an exhibit of decided skill that reflects great credit upon the inventor. He also read a paper describing the effects of steam, and of dry heat, or *super-dry* heat, on rubber and celluloid, and followed with an explanation of the working qualities of his new appliance, which he claims as the safest and, in all respects, by far the best in use.

